# Avocado Consumption Alters Gastrointestinal Bacteria Abundance and Microbial Metabolite Concentrations among Adults with Overweight or Obesity: A Randomized Controlled Trial

**DOI:** 10.1093/jn/nxaa219

**Published:** 2020-08-17

**Authors:** Sharon V Thompson, Melisa A Bailey, Andrew M Taylor, Jennifer L Kaczmarek, Annemarie R Mysonhimer, Caitlyn G Edwards, Ginger E Reeser, Nicholas A Burd, Naiman A Khan, Hannah D Holscher

**Affiliations:** Division of Nutritional Sciences, University of Illinois, Urbana-Champaign, IL, USA; Division of Nutritional Sciences, University of Illinois, Urbana-Champaign, IL, USA; Department of Food Science and Human Nutrition, University of Illinois, Urbana-Champaign, IL, USA; Division of Nutritional Sciences, University of Illinois, Urbana-Champaign, IL, USA; Department of Food Science and Human Nutrition, University of Illinois, Urbana-Champaign, IL, USA; Division of Nutritional Sciences, University of Illinois, Urbana-Champaign, IL, USA; Department of Kinesiology and Community Health, University of Illinois, Urbana-Champaign, IL, USA; Division of Nutritional Sciences, University of Illinois, Urbana-Champaign, IL, USA; Department of Kinesiology and Community Health, University of Illinois, Urbana-Champaign, IL, USA; Division of Nutritional Sciences, University of Illinois, Urbana-Champaign, IL, USA; Department of Kinesiology and Community Health, University of Illinois, Urbana-Champaign, IL, USA; Neuroscience Program, University of Illinois, Urbana-Champaign, IL, USA; Division of Nutritional Sciences, University of Illinois, Urbana-Champaign, IL, USA; Department of Food Science and Human Nutrition, University of Illinois, Urbana-Champaign, IL, USA; Department of Kinesiology and Community Health, University of Illinois, Urbana-Champaign, IL, USA; National Center for Supercomputing Applications, University of Illinois, Urbana-Champaign, IL, USA; Institute of Genomic Biology, University of Illinois, Urbana-Champaign, IL, USA

**Keywords:** microbiota, adiposity, dietary fiber, bile acids, short-chain fatty acids, branched-chain fatty acids

## Abstract

**Background:**

Avocados are rich in dietary fiber and monounsaturated fatty acids (MUFAs), nutrients that have been independently connected to metabolic health benefits and the gastrointestinal microbiota.

**Objectives:**

We aimed to evaluate the impact of avocado consumption on the gastrointestinal microbiota and microbial metabolites, secondary outcomes of the *Persea americana* for Total Health (PATH) study, and conduct exploratory analyses to assess relations between the fecal microbiota, fecal metabolites, and health markers.

**Methods:**

Adults [*n* = 163, 25–45 y, BMI (kg/m^2^) ≥ 25.0] were enrolled in the PATH study, a 12-wk investigator-blinded trial where participants were batch randomized to match the 2 groups by age, sex, visceral adiposity, and fasting glucose concentrations. Participants consumed isocaloric meals with or without avocado (175 g, men; 140 g, women) once daily for 12 wk. The fecal microbiota was assessed with 16S ribosomal RNA gene (V4 region) sequencing and analysis using DADA2 and QIIME2. Fecal fatty acid and bile acid concentrations were quantified using GC and LC-MS. Per-protocol (≥80% meal consumption) and intent-to-treat analyses were conducted using univariate ANOVA and Mann-Whitney *U* tests. Bivariate correlations were conducted between fecal microbiota, fecal metabolites, and health measures.

**Results:**

The avocado treatment increased ɑ diversity and enriched *Faecalibacterium, Lachnospira*, and *Alistipes* between 26% and 65% compared with the control group. The avocado group had 18% greater fecal acetate, 70% greater stearic acid, and 98% greater palmitic acid concentrations than the control group, while the concentrations of the bile acids cholic and chenodeoxycholic acid were 91% and 57% lower, respectively.

**Conclusions:**

Daily avocado consumption resulted in lower fecal bile acid concentrations, greater fecal fatty acid and SCFAs, and greater relative abundances of bacteria capable of fiber fermentation, providing evidence that this nutrient-dense food affects digestive physiology, as well as the composition and metabolic functions of the intestinal microbiota. This trial was registered at www.clinicaltrials.gov as NCT02740439.

## Introduction

Overweight and obesity impact a majority of Americans (1, 2). Connections between excess adiposity and an altered community of gastrointestinal microorganisms are apparent, but conflicting findings remain regarding obesity-related differences in the microbiota (3–5) and microbial and metabolic responses to dietary interventions among individuals with overweight and obesity. Volatile fatty acids are microbially derived metabolites that include short-chain fatty acids (SCFAs) and branched-chain fatty acids (BCFAs). These metabolites are produced through microbial metabolism of carbohydrates and proteins that are mostly obtained from the diet (6). However, while SCFAs are commonly considered beneficial for metabolic health (7), concentrations of these metabolites are often greater among those with overweight and obesity (8). Therefore, more research is necessary to delineate the impact of utilizing dietary approaches to modulate the gastrointestinal microbiota and volatile fatty acid concentrations to influence metabolic health in individuals with overweight and obesity.

Bile acids are steroid molecules that are formed in the liver, stored in the gallbladder, and released into the intestinal lumen for dietary fat emulsification (9). Bile acids can alter gastrointestinal microbial communities, as well as be modified by microorganisms in the gut (9). A Western diet higher in total and saturated fat increases the bile acid pool size (10, 11). Greater bile acid concentrations, in particular the hydrophobic secondary bile acids deoxycholic and lithocholic acid, act as antimicrobial agents that induce intestinal inflammation and an expansion of bile-tolerant microbes linked to deleterious health effects (12). The bile acid pool composition can be influenced by diet–microbiota interactions and subsequently alter gut health and immunity, making diet-induced modifications in bile acid profiles a potential strategy for improving the health of individuals with overweight and obesity.

Avocados are rich in fiber and MUFAs (13, 14). Regular avocado consumption is associated with lower body weight (15, 16). Avocado interventions from 1 to 12 wk revealed increased satiety and reductions in blood lipid concentrations (17–21). A preclinical trial reported increased fecal acetate concentrations in rats fed an avocado diet versus control; however, there were no differences between groups in the fecal microbiota (22). Conversely, a 12-wk hypocaloric intervention, which involved avocado, reported microbial differences between the avocado and control groups (23). However, the impact of avocado consumption on gut bacteria and metabolites in the absence of caloric restriction is unknown.

The objectives of this study were to evaluate the impact of daily intake of avocado on the fecal microbiota and microbial metabolites, secondary outcomes of the study, and to assess relations between metabolic health markers, microbial taxa, and metabolites in adults with overweight and obesity. We hypothesized that *1*) avocado consumption would alter the fecal bile acid and fatty acid pool and increase the relative abundance of microorganisms capable of fiber degradation and SCFA production as compared with control and *2*) fecal bacterial abundances and microbially derived metabolites would be related to metabolic health outcomes. Elucidating the impact of avocado intake on the intestinal microbiota is anticipated to be a translatable dietary strategy to improve health and alter the gut microbiota among adults with overweight and obesity.

## Methods

### Study design and participant eligibility

The *Persea americana* for Total Health (PATH) study, was an investigator-blinded, parallel-arm, randomized controlled trial of a 12-wk dietary intervention. Participants were batch randomized to match the 2 groups on age, sex, visceral adiposity, and fasting glucose concentrations using an automated algorithm in SAS (SAS Institute Inc., Cary, NC). Randomization was conducted by a research staff member not involved in data collection. The primary objective of the PATH study was to assess glycemic control and abdominal adiposity. Herein, we report measures of the intestinal microbiota and microbially derived metabolites, which were secondary outcomes of the PATH study. Exploratory, correlational analyses were also conducted between bacteria significantly affected by avocado intake and measures of metabolic health in the per-protocol avocado group participants studied in the current report.

Adults between 25 and 45 y of age with a BMI (kg/m^2^) ≥25.0 were enrolled in this study. Participants were recruited from the central Illinois area through e-mail, print, and word-of-mouth advertising. Study exclusion criteria included the following: *1*) BMI <25.0, *2*) pregnancy or lactating, *3*) current tobacco use, *4*) previous diagnosis of metabolic or gastrointestinal disease, *5*) food allergies or intolerances, *6*) use of medications that impact normal bowel function, or *7*) malabsorptive or restrictive bariatric surgery within the previous 2 y. All participants provided written informed consent before study initiation. Study procedures were administered in accordance with the Declaration of Helsinki and were approved by the University of Illinois Institutional Review Board. This trial is registered at www.clinicaltrials.gov as NCT02740439.

### Dietary intervention

Participants received 175 g (men) or 140 g (women) of fresh Hass avocado daily as part of a meal or an isocaloric control meal once per day for 12 wk. Meals were provided based on a 7-d menu cycle that incorporated standard ingredients commonly consumed by American adults. Meal ingredients were >90% similar between the avocado and control groups and identical with regard to energy and macronutrient composition (**Table 1**). The study meals were designed to replace 1 meal/d. Female participants received meals that provided 20% less energy than the meals provided to male participants. Meals comprised approximately one-third of mean male-estimated energy requirements (1970 ± 289 kcal; range: 1370–2560 kcal) and female-estimated energy requirements (1720 ± 353 kcal; 980–3040 kcal) (**Supplemental Methods**). Macronutrient percentages within study meals were within the Acceptable Macronutrient Distribution Ranges (24). Compared with the control meals, the avocado meals were designed to contain 10% less energy/d from SFAs, 14% in additional energy from MUFAs, and 12 g of additional dietary fiber. Meal compilation occurred in a metabolic kitchen, and each ingredient was weighed to the nearest gram. Participants traveled to the test site twice weekly to obtain study meals. Insulated meal coolers, ice packs, and information about food safety procedures were provided. Participants were instructed to replace 1 meal/d (i.e., breakfast, lunch, or dinner) with the provided study meals to provide variability in offerings to enhance compliance. Intervention compliance was evaluated by analysis of daily self-report compliance records that were delivered to investigators at each meal pick-up appointment.

**TABLE 1:** Nutrient and food-group compositions of the control and avocado meals that were provided to adult participants daily for 12 wk

	Control	Avocado
Nutrient		
Energy, kcal/d		
Men	660	662
Women	538	530
Total fat, %	39	40
SFAs	17	7
MUFAs	10	24
PUFAs	9	5
Carbohydrate, %	45	45
Protein, %	16	14
Total fiber, g/d	4	16
Soluble fiber	1	6
Pectins	0	4
Insoluble fiber	3	10
Lutein + zeaxanthin, μg	205	701
Food groups, g/d		
Fruits	33	455
Vegetables	195	254
Grain	76.7	59.6
Meat	31.2	31.2
Dairy	196	73.5
Fats and oils	78.4	8.4

### Dietary intake

Habitual dietary intake was evaluated using 7-d diet records. Participants were instructed by a trained research staff member to record all food and beverages consumed over a 7-d period at baseline and prior to post-testing at week 12. Food diaries included detailed instructions and figures to assist with portion-size approximation. Seven-day diet records were subsequently entered into and analyzed using Nutrition Data System for Research (NDSR) version 2015 software (Nutrition Coordinating Center, University of Minnesota, Minneapolis, MN).

### Weight status and adiposity

Height (centimeters) and weight (kilograms) were measured in triplicate using a Seca model 240 stadiometer (Seca, Hamburg, Germany) and a Tanita WB-300 Plus digital scale (Tanita Corporation, Tokyo, Japan), respectively. BMI was calculated as kg/m^2^. Adiposity was assessed using a DXA Hologic Horizon W bone densitometer using the standard software (APEX Software version 5.6.0.5; Hologic, Bedford, MA).

### Fecal microbiota analysis

Fecal samples were homogenized, placed in aliquots, flash frozen, and stored at −80°C. Fecal DNA was isolated and the V4 region of the 16S ribosomal RNA gene was amplified then sequenced at the WM Keck Biotechnology Center as previously described (25). Sequences were demultiplexed in Quantitative Insights Into Microbial Ecology version 2 (QIIME2) version 2019.4, and amplicon sequence variants were generated using the DADA2 version 1.10.1 denoise-single plugin using default settings following dereplication and standard quality-filtering procedures (e.g., the removal of sequencing-related barcodes, sequences with quality scores <20, and chimeric sequences) (26–28). Sequences were rarified at 6450 (intent-to-treat) and 6652 (per protocol) sequences per sample prior to ɑ- and β-diversity analysis. ɑ-Diversity was assessed with the Faith Phylogenetic Diversity metric or the quantification of taxon richness through phylogenetic tree units (29). β-Diversity was evaluated with permutational multivariate analysis of variance (PERMANOVA) weighted Unifrac distances between samples (30). Taxonomy was assigned using the q2-feature-classifier command with default parameters in QIIME2 and sequences were matched against the Greengenes 13_8 database (31). Differential abundance comparisons between groups was performed using nonrarified sequence data.

### Fecal metabolites

Participants provided fecal samples within 15 min of defecation. Samples were collected by participants at home or work and delivered to the study site. Participants were encouraged to utilize lavatories near study sites if transportation was likely to exceed the 15-min drop-off window. Fecal aliquots for volatile fatty acid (C ≤ 6) analysis were weighed, acidified with 2 N HCl, and stored at −20°C until analysis. SCFA (butyrate, propionate, and acetate) and BCFA (isobutyrate, valerate, and isovalerate) concentrations were quantified using GC-LC (180 cm × 4 mm i.d. glass column with 10% SP-1200/1% HVFA H_3_PO_4_ on 80/100 mesh Chromosorb WAW; Hewlett-Packard 5890A Series II gas chromatograph; Supelco, Inc., Bellefonte, PA) and normalized on a dry matter basis (μmol/g) (32, 33).

Fecal fatty acid extraction was performed for a subset of study participants (*n* = 45) at the Roy G Carver Metabolomics Center based on previously published protocols (34, 35). Metabolite profiles were generated using a gas chromatograph (Agilent 7890A; Agilent, Inc., Palo Alto, CA), a mass selective detector (Agilent 5975), and autosampler (Agilent 7683B). Chromatogram peaks were then compared with electron impact mass spectrum libraries [in-house custom library; NIST08 (NIST, Gaithersburg, MD); WILEY08 (Palisade Corporation, Ithaca, NY)] and evaluated using MSD ChemStation (Agilent, Palo Alto, CA) and AMDIS (NIST) programs for metabolite identification. Fecal fatty acid concentrations were normalized by sample weight and are presented as relative concentrations per 100 mg fecal matter.

Unconjugated primary bile acids cholic acid (3α, 7α, 12α-trihydroxy-5β-cholan-24-oic acid) and chenodeoxycholic acid (3α, 7α-dihydroxy-5β-cholan-24-oic acid) and secondary bile acids deoxycholic acid (3α, 12α-dihydroxy-5β-cholan-24-oic acid) and lithocholic acid (3α-hydroxy-5β-cholan-24-oic acid) were quantified via LC-MS/MS as previously described (36). Lyophilized fecal samples were suspended in sodium acetate buffer (0.5 mM, pH 5.6), refluxed in ethanol, centrifuged, and applied to a 500-mg/6-mL C18 cartridge. A sample aliquot was combined with ethanol and internal standard (2,2,4,4-d4 cholic acid in 50% ethanol at 200 pmol/μL) and injected into an LC-MS/MS system (Sciex 550 QTrap, Agilent 1200 LC) at the WM Keck Biotechnology Center.

### Statistical analyses

Data were analyzed using SPSS (version 25; IBM, Armonk, NY) and QIIME2 version 2019.4. Variables were evaluated for normality using the Shapiro-Wilk test. Microbial taxa and metabolic biomarkers were transformed as needed using arcsine and natural log transformations, respectively (37). Baseline differences in demographic and metabolic health variables were evaluated by sex using chi-square or independent *t* test. Baseline fecal microbiota and metabolite data were also assessed using Mann-Whitney *U* (38, 39) or independent *t* tests. Study outcomes were analyzed by treatment group at 12 wk post-treatment. The intent-to-treat participant group included those with available metabolic health data at post-test and carried-forward baseline fecal microbiota observations for participants who dropped out of the study. Per-protocol analyses were conducted for participants meeting ≥80% of meal consumption at the 12-wk follow-up. Univariate generalized linear modeling procedures or Mann-Whitney *U* tests (38, 39) were applied to assess post-test differences in bacteria relative abundances and fecal metabolites by treatment group. Bacteria abundances were adjusted for multiple comparisons using the Benjamini-Hochberg false discovery rate (FDR) method (40). Spearman's ρ and Pearson's bivariate correlations were utilized to evaluate relations between fecal bacteria found to differ significantly between the treatment groups at follow-up, fecal metabolites, and metabolic health outcomes. *P* values ≤0.05 were considered statistically significant and values between 0.05 and 0.10 were considered trends.

## Results

### Participant characteristics

**FIGURE 1:**
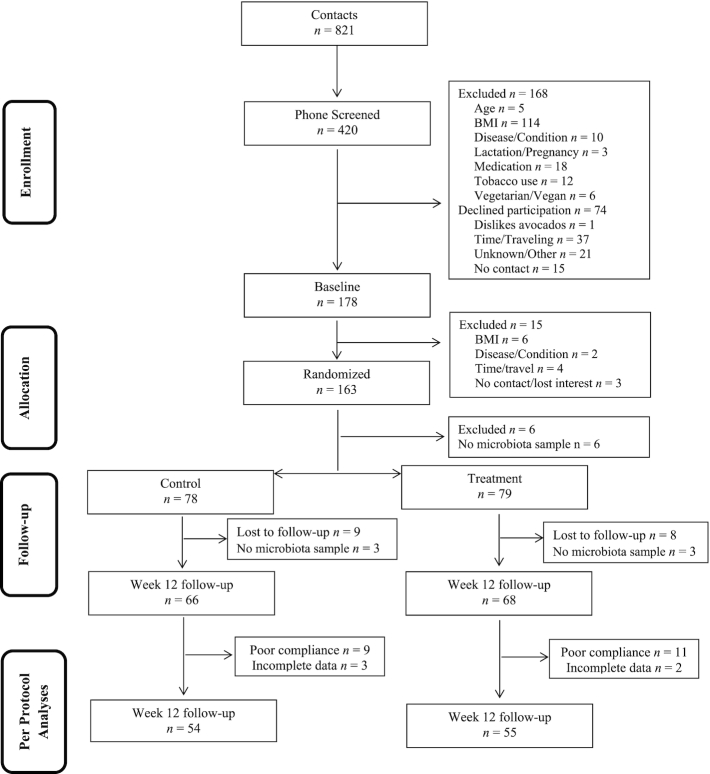
PATH study CONSORT diagram for participants with microbiota data. CONSORT, Consolidated Standards of Reporting Trials; PATH, *Persea americana* for Total Health.

**Figure 1** describes study recruitment and inclusion/exclusion criteria. Of the 178 eligible participants, 163 were randomly assigned and 157 provided baseline fecal samples that were included in the intent-to-treat fecal microbiota and metabolite analyses. Study outcomes were also analyzed for the 109 per-protocol participants at the 12-wk follow-up time point who provided fecal samples at both baseline and endline. Diet records were analyzed for 106 per-protocol participants who provided ≥5 d of information. Dietary record data revealed that baseline avocado intake was low (0.10 ± 0.01 servings/d) and less than half of participants reported any avocado consumption at baseline. Control participants reported consuming 0.04 $ \pm 0.02$ servings/d of avocado at 12 wk, which was not different from baseline. Conversely, the avocado treatment group reported consuming significantly more avocado at the 12-wk timepoint (2.3 $ \pm 0.4$ servings/d; *P* < 0.001). Self-reported study meal intake was 90% ± 1% during the 12-wk intervention and most participants (*n* = 133, 82%) reported meal consumption of ≥80%. Participants (**Table 2**) were predominantly female (64%) and of Caucasian descent (77%). Forty-three percent of participants had overweight and 57% had obesity. Female participants presented with significantly larger BMI values (*P* = 0.02), and 68% of this group presented with obesity. Male participants were predominantly (61%) in the overweight BMI category.

**TABLE 2:** Participant characteristics at baseline^1^

	All		Women		Men		
	Values	*n*	Values	*n*	Values	*n*	*P*
Age,^2^ y	35 ± 0.5	157	35 ± 0.6	100	35 ± 0.8	57	0.86
Race,^3^ %							0.15
American Indian or Alaskan Native	1	1	0	0	1	2	
Asian	14	10	7	8	7	13	
African American	10	7	6	7	4	8	
White	105	77	68	82	37	70	
Mixed race	6	4	2	2	4	8	
Hispanic identity,^3^ %	11	7	6	6	5	9	0.10
Body weight,^2^ kg	95.4 ± 1.63	157	92.4 ± 1.99	78	100.0 ± 3.15	51	0.04
BMI,^2^ kg/m^2^	32.8 ± 0.5	154	33.7 ± 0.6	97	31.3 ± 0.9	57	0.02
Overweight (25.0–29.9 kg/m^2^),^3^ %	66	43	31	32	35	61	0.01
Obese,^3^ %							
30.0–34.9 kg/m^2^	42	27	31	32	11	19	
35.0–39.9 kg/m^2^	23	15	18	19	5	9	
≥40 kg/m^2^	23	15	17	18	6	11	
Visceral abdominal adiposity,^2^ g	681 ± 23.8	151	694 ± 27.9	96	658 ± 44.0	55	0.47
Subcutaneous abdominal adiposity,^2^ g	2.15 × 10^3^ ± 60.3	151	2.43 × 10^3^ ± 62.2	96	1.67 × 10^3^ ± 94.6	55	<0.0001

1 Values are as means ± SEMs or counts and percentages. Participant characteristics were analyzed using independent *t* test or chi-square test to compare differences by sex.

^2^Independent *t* test.

^3^Chi-square test.

### Per-protocol results

#### Dietary intake and body weight at 12-wk follow-up

As intended, participants in the avocado group reported MUFA consumption that was ∼20 g greater than the control group and 14 g of additional dietary fiber. Self-reported total energy intake tended to be 300 kcal/d higher in the avocado compared with the control group (*P* = 0.07) (**Table 3**). Interestingly, dietary record results revealed similar saturated and polyunsaturated fat intake between the 2 groups, despite the differences in these fatty acids between the control and intervention meals. Body weight did not differ between groups at 12-wk follow-up (control group, 96.0 ± 2.68 kg; avocado group, 96.2 ± 2.76 kg; *P* = 0.95).

**TABLE 3:** Per-protocol analysis comparisons at 12-wk follow-up of dietary intake between groups of adults who consumed a daily isocaloric meal with or without avocado^1^

	Control (*n* = 52)	Avocado (*n* = 54)	*P*
Total energy, kcal/d	2170 ± 83	2500 ± 167	0.07
Total fat, g/d	89.5 ± 3.6	112.0 ± 8.0	0.01
Saturated fat, g/d	33.0 ± 1.4	32.4 ± 2.0	0.40
Monounsaturated fat, g/d	28.9 ± 1.3	48.2 ± 4.3	<0.001
Polyunsaturated fat, g/d	20.4 ± 1.0	20.9 ± 1.4	0.81
Total carbohydrate, g/d	247 ± 10.9	274 ± 18.3	0.19
Total protein, g/d	83.8 ± 3.2	96.8 ± 7.6	0.17
Total dietary fiber, g/d	17.3 ± 1.0	31.4 ± 3.3	<0.001
Insoluble dietary fiber, g/d	11.2 ± 0.7	20.0 ± 2.1	<0.001
Soluble dietary fiber, g/d	5.8 ± 0.3	10.9 ± 1.1	<0.001
Pectins, g/d	2.0 ± 0.1	6.0 ± 0.8	<0.001

1 Values are means ± SEMs. Data were included for participants with ≥5 d of diet records. Diet records were analyzed by univariate ANOVA.

#### Fecal microbiota and metabolites

**FIGURE 2:**
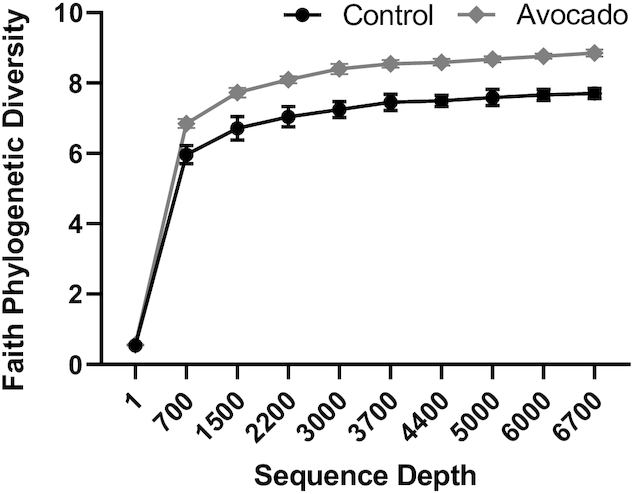
Per-protocol analysis comparisons at 12-wk follow-up of Faith Phylogenetic Diversity between groups of adults who consumed a daily isocaloric meal with or without avocado. ɑ-Diversity was assessed using the Faith Phylogenetic Diversity metric following rarefaction at 6652 sequences per sample. Values are means ± SEMs, *n* = 54 (control) and *n* = 55 (avocado).

**FIGURE 3:**
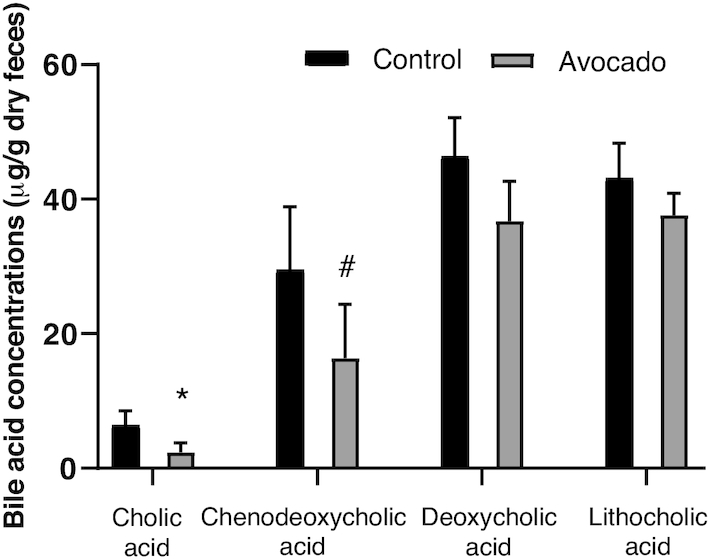
Per-protocol analysis comparisons at 12-wk follow-up of fecal primary and secondary bile acid concentrations between groups of adults who consumed a daily isocaloric meal with or without avocado. Data were analyzed by univariate ANOVA and are presented as means ± SEMs of the mean (*n* = 44, control; *n* = 46, avocado). *Different from control, *P* ≤ 0.05; ^#^tended to differ from control *P* < 0.1.

**FIGURE 4:**
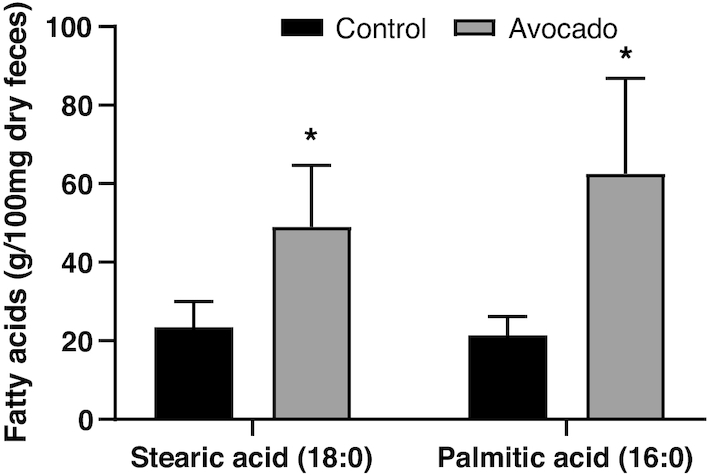
Per-protocol analysis comparisons at 12-wk follow-up of fecal fatty acid concentrations between groups of adults who consumed a daily isocaloric meal with or without avocado. Data were analyzed by univariate ANOVA and are presented as means ± SEMs of the mean (*n* = 19, control; *n* = 27, avocado). *Different from control, *P* ≤ 0.05.

Faith Phylogenetic Diversity, a measure of microbiota α-diversity, was greater among the avocado group (*P* = 0.02) (**Figure 2**). The weighted Unifrac distances, a measure of β-diversity, tended to be greater in the avocado group compared with the control group at the 12-wk follow-up visit (*P* = 0.09) (**Supplemental Figure 1**). At the genus level, the relative abundances of *Faecalibacterium, Lachnospira*, and *Alistipes* were enriched in the avocado group, while the relative abundances of *Roseburia* and *Ruminococcus* were diminished (**Table 4**). Fecal acetate (*P* = 0.01) concentrations were greater in the avocado group as compared with the control group at the end of the intervention period (**Table 5**). Additionally, concentrations of cholic acid (*P* = 0.01) were greater and chenodeoxycholic acid tended to be higher (*P* = 0.07) in the control group (**Figure 3**; **Supplemental Table 1**), whereas concentrations of a variety of SFAs and MUFAs were greater in the avocado group (**Figure 4**; **Supplemental Table 2**).

**TABLE 4:** Per-protocol analysis comparisons at 12-wk follow-up of the relative abundance of fecal bacteria between groups of adults who consumed a daily isocaloric meal with or without avocado^1^

	Control (*n* = 54)	Avocado (*n* = 55)	*P*	*q*
Firmicutes^2^	59.9 ± 2.05	58.8 ± 1.98	0.71	0.80
*Blautia*^2^, ^3^	4.48 ± 0.65	3.64 ± 0.43	0.34	0.63
*Coprococcus*^2^	3.54 ± 0.33	3.66 ± 0.31	0.78	0.84
*Ruminococcus*^2^, ^3^	3.22 ± 0.37	2.22 ± 0.29	0.02	0.28
*Dorea*^2^, ^3^	1.00 ± 0.12	1.04 ± 0.12	0.70	0.82
*Streptococcus*^4^	0.47 ± 0.10	0.75 ± 0.23	0.90	0.93
*Faecalibacterium*^2^	13.0 ± 1.12	16.4 ± 1.25	0.04	0.28
*Roseburia*^2^	9.24 ± 0.93	6.12 ± 0.62	0.01	0.28
*Clostridium*^2^, ^3^	0.36 ± 0.07	0.46 ± 0.06	0.08	0.32
*Oscillospira*^2^, ^3^	1.07 ± 0.15	1.17 ± 0.16	0.43	0.75
*Dialister*^2^, ^3^	2.33 ± 0.39	2.05 ± 0.39	0.30	0.65
*Lactobacillus*^4^	0.14 ± 0.08	0.15 ± 0.08	0.20	0.56
*Lachnospira*^2^	1.12 ± 0.13	1.56 ± 0.17	0.04	0.22
*Lachnobacterium*^4^	0.32 ± 0.11	0.14 ± 0.07	0.20	0.51
*Phascolarctobacterium*^2^, ^3^	0.66 ± 0.15	0.62 ± 0.12	0.62	0.79
*Alistipes*^2^, ^3^	1.44 ± 0.23	2.37 ± 0.45	0.03	0.28
Bacteroidetes^2^	34.5 ± 2.11	34.3 ± 1.96	0.94	0.94
*Bacteroides*^2^	24.5 ± 2.09	22.8 ± 1.87	0.55	0.86
*Parabacteroides*^2^	1.92 ± 0.29	2.73 ± 0.29	0.05	0.23
Actinobacteria^2^, ^3^	1.98 ± 0.40	2.32 ± 0.42	0.56	0.83
*Bifidobacterium*^2^, ^3^	1.38 ± 0.32	1.54 ± 0.30	0.45	0.74
*Collinsella*^4^	0.36 ± 0.20	0.39 ± 0.16	0.57	0.80
Verrucomicrobia^4^	0.63 ± 0.17	0.98 ± 0.26	0.08	0.28
* Akkermansia* ^4^	0.63 ± 0.17	0.96 ± 0.26	0.15	0.47
Proteobacteria^2^	2.51 ± 0.29	2.70 ± 0.29	0.65	0.79
*Sutterella*^2^, ^3^	1.49 ± 0.20	1.72 ± 0.25	0.57	0.76
*Bilophila*^2^, ^3^	0.23 ± 0.04	0.31 ± 0.06	0.23	0.54
*Desulfovibrio*^4^	0.10 ± 0.04	0.15 ± 0.05	0.32	0.64

1 Values are means ± SEMs. Data were analyzed by univariate ANOVA or Mann-Whitney *U* test with false discovery rate correction (*q* values).

^2^ANOVA.

^3^Indicates bacteria that were transformed prior to analysis.

^4^Mann-Whitney *U* test.

**TABLE 5:** Per-protocol analysis comparisons at 12-wk follow-up of fecal volatile fatty acid concentrations between groups of adults who consumed a daily isocaloric meal with or without avocado^1^

Volatile fatty acid	Control, μmol/g DM feces (*n* = 52)	Avocado, μmol/g DM feces (*n* = 54)	*P*
Acetate^2^	260 ± 15.0	311 ± 20.4	0.04
Propionate^2^	81.5 ± 6.03	75.1 ± 7.66	0.14
Butyrate^2^	65.5 ± 5.85	57.2 ± 5.33	0.30
Summed SCFAs^2^	385 ± 21.1	437 ± 25.6	0.35
Isobutyrate^2^	7.96 ± 0.55	8.18 ± 0.49	0.81
Isovalerate^2^	8.66 ± 0.63	8.68 ± 0.56	0.98
Valerate^3^	9.08 ± 1.04	7.86 ± 0.55	0.17
Summed BCFAs^2^	25.7 ± 1.96	24.7 ± 1.31	0.68
SCFA:BCFA ratio^3^	23.7 ± 6.49	18.6 ± 1.15	0.45

1 Values are means ± SEMs. Data were analyzed by univariate ANOVA or Mann-Whitney *U* test^.^ BCFA, branched-chain fatty acid; DM, dry matter.

^2^ANOVA.

^3^Mann-Whitney *U* test.

#### Associations between fecal bacteria, metabolites, and metabolic outcomes

Correlational analyses were conducted within the avocado group among bacteria that differed by group at the end of the intervention and fecal metabolites and metabolic health outcomes. Inverse relations were observed between *Lachnospira* and fecal cholic acid concentrations (*r* = −0.32, *P* = 0.05, *n* = 40), insulin AUC (*r* = −0.36, *P* = 0.02, *n* = 45), and TNF-$\alpha $ concentrations (*r* = −0.32, *P* = 0.02, *n* = 50) in the avocado group (Supplemental Methods; **Table 6**). *Alistipes* was also negatively related to fecal cholic acid concentrations (*r* = −0.35, *P* = 0.03, *n* = 40) in the avocado group.

**TABLE 6:** Per-protocol analysis of the associations between fecal bacteria, fecal metabolites, and metabolic outcomes at the 12-wk follow-up in adults who consumed a daily isocaloric meal with avocado^1^

		*Roseburia*		*Ruminococcus*		*Lachnospira*		*Alistipes* ^2^		*Faecalibacterium*	
	*n*	*r*	*P*	*ρ*	*P*	*r*	*P*	*r*	*P*	*r*	*P*
Acetate, μmol/g DM feces	54	0.13	0.36	0.02	0.87	−0.06	0.65	−0.26	0.06	0.24	0.08
Cholic acid, μmol/g dry feces,	40	−0.29	0.07	−0.05	0.74	−0.29	0.07	−0.35	0.03	0.04	0.79
Chenodeoxycholic acid, μmol/g dry feces	36	−0.10	0.56	−0.11	0.54	−0.23	0.18	−0.20	0.23	0.13	0.45
Lithocholic acid, μmol/g dry feces	45	0.05	0.72	−0.11	0.48	0.12	0.43	0.06	0.70	−0.06	0.69
Deoxycholic acid, μmol/1 g dry feces	43	−0.19	0.23	−0.16	0.31	−0.12	0.46	−0.20	0.20	−0.07	0.64
Plasma glucose AUC, mmol/L × 120 min	46	−0.09	0.55	−0.05	0.76	−0.09	0.56	−0.16	0.30	−0.08	0.60
Plasma insulin AUC, pmol/L × 120 min	45	0.08	0.60	−0.27	0.08	−0.32	0.03	−0.13	0.38	−0.02	0.88
HOMA-IR	45	−0.02	0.91	−0.14	0.35	−0.08	0.61	−0.09	0.57	0.04	0.79
Matsuda index	44	−0.15	0.35	0.26	0.09	0.19	0.22	0.14	0.37	−0.05	0.77
Plasma TNF-ɑ$,$ pg/mL	50	−0.04	0.80	−0.02	0.92	−0.15	0.29	−0.09	0.53	0.00	0.98
Plasma CRP, μg/mL	49	0.13	0.37	−0.12	0.42	−0.17	0.23	0.22	0.13	0.15	0.32
Plasma IL-6, pg/mL	49	0.09	0.54	−0.03	0.83	−0.08	0.60	0.23	0.11	0.12	0.42
Visceral abdominal adiposity, g	53	−0.14	0.33	−0.27	0.05	−0.09	0.52	0.10	0.47	−0.12	0.38
Subcutaneous abdominal adiposity, g	53	−0.02	0.87	0.00	0.99	−0.10	0.48	0.26	0.06	0.03	0.84

1 Data were analyzed by Spearman or Pearson bivariate correlations. CRP, C-reactive protein; HOMA-IR, homeostatic model assessment of insulin resistance; IL-6, interleukin-6; TNF-ɑ, tumer necrosis factor ɑ; .

^2^Data were transformed prior to analysis.

### Intent-to-treat analyses

#### Dietary intake and body weight at 12-wk follow-up

As intended, participants in the avocado group reported greater intake of MUFAs, total dietary fiber, insoluble dietary fiber, soluble dietary fiber, and pectin. Saturated fat, polyunsaturated fat, total carbohydrate, and total protein intakes were similar between groups (**Supplemental Table 3**). Between-group body weight was similar at 12-wk follow-up (control group, 94.7 ± 2.21 kg; avocado group, 95.5 ± 2.37 kg; *P* = 0.80).

#### Fecal microbiota and metabolites

Weighted Unifrac distances, a measure of microbiota β-diversity, tended to be different between groups (*P* = 0.053) (**Supplemental Figure 2**). Faith Phylogenetic Diversity, a measure of α-diversity, did not differ between groups (*P* = 0.13). The relative abundances of *Alistipes* (*P* = 0.003) and *Ruminococcus* (*P* = 0.01) were greater and *Faecalibacterium* (*P* = 0.07) and *Lachnospira* tended to be enriched (*P* = 0.06) at the end of the 12-wk intervention period in the avocado group (**Supplemental Table 4**). *Lachnobacterium* (*P* = 0.08) tended to be greater in the control group.

Fecal acetate concentrations (*P* = 0.02) were greater in the avocado group when compared with the control group (**Supplemental Figure 3**). Differences in fecal bile acid concentrations (**Supplemental Figure 4, Supplemental Table 5**) were also evident, including greater fecal cholic acid concentrations (*P* = 0.01) in the control group. The control group tended to have greater fecal deoxycholic acid (*P* = 0.08), lithocholic acid (*P* = 0.09), summed primary bile acid concentrations (*P* = 0.07), and total bile acid concentrations (*P* = 0.07).

## Discussion

The present study evaluated fecal microbiota and metabolite concentrations following a randomized controlled trial where adults with overweight and obesity consumed a daily meal with or without avocado for 12 wk. Avocado consumption resulted in differences in fecal bacteria and microbial metabolite concentrations, changes that were more pronounced among participants with high treatment compliance (i.e., among per-protocol participants). In the avocado group who met per-protocol criteria, we observed greater α-diversity; enrichment of *Faecalibacterium, Lachnospira*, and *Alistipes* relative abundances; and an 18% between-group increase in fecal acetate concentrations. Despite reporting higher total fat intake, the per-protocol avocado group had diminished fecal bile acid concentrations, including 91% and 57% lower cholic acid and chenodeoxycholic acid concentrations, respectively, and numerically lower concentrations of the secondary bile acids deoxycholic (23%) and lithocholic (14%) acid.

The increase in α-diversity with the avocado treatment among per-protocol participants likely indicates that more frequent avocado consumption may be necessary to elicit a measurable effect on microbiota diversity. These observed differences may also be related to differences in study meal dietary fat composition and subsequent fecal fatty acid and bile acid profile changes. Indeed, fatty acid excretion was enhanced in a subset of participants in the avocado group, while cholic, chenodeoxycholic, deoxycholic, and lithocholic acid concentrations were reduced. Gastrointestinal microorganisms, as well as dietary fat and fiber intake, are implicated in bile acid regulation. For example, *Clostridium* spp. and *Eubacterium* spp. have the enzymatic capacity to modify bile acids (41). Total fat and saturated fat consumption increases total fecal bile acid concentrations (10), while consumption of soluble, viscous dietary fiber increases fecal fat excretion (42–46). Insoluble fiber also reduces total fecal bile acid concentrations through an increase in fecal weight (47). In preclinical studies, saturated fat intake reduced microbial diversity (48) and increased bile acid synthesis (49), and subsequent intestinal inflammation (11). Conversely, high-MUFA diets exert antimicrobial activity (10) and ameliorate high-fat-diet–related reductions in microbial diversity (50). Clinical studies revealed high-fiber diets increase fecal weight (51) and 42 g/d of walnuts, a rich source of MUFA, reduced fecal secondary bile acids deoxycholic and lithocholic acid by 29% and 64%, respectively, compared with control (25). A 12-wk study reported that 15 g pea fiber/d reduced fecal cholic and deoxycholic acid concentrations by 25% and 48%, respectively (52). A Hass avocado (175-g serving) contains 42% of the Daily Value for total fat at 27 g but is rich in both MUFAs (17 g) and dietary fiber (12 g). To our knowledge, this study is the first to report the impact of avocado intake on fecal fatty acid and bile acid concentrations among adults.

The relative abundances of *Faecalibacterium* and *Lachnospira* were enriched in the avocado group compared with the control group. Interestingly, *Lachnospira* was negatively related to fecal cholic acid concentrations and plasma insulin AUC among per-protocol participants. *Faecalibacterium* is a bile-sensitive genus (53) that, via the action of butyl-CoA, can convert 2 acetate molecules to butyrate and is found in lower abundance among those with obesity (54) and metabolic dysfunction (55). The reduction in fecal bile acid concentrations with avocado intake in the current study may have supported the observed bloom in *Faecalibacterium*. A 6-mo controlled-feeding trial among healthy adults reported that a diet with a similar total fat content to the present study (40% of total energy) reduced *Faecalibacterium* relative abundances when compared with moderate-fat (30% of energy) or low-fat (20% of energy) diets (56). However, the intervention diet fat content was predominantly composed of PUFAs (11–24%), with only 5–9% of energy derived from MUFAs, indicating that dietary fat composition, rather than fat quantity, may be responsible for these findings. Further work is necessary to elucidate the causal impact of dietary changes on these taxa and subsequent metabolic health outcomes among adults with overweight and obesity.

Avocado consumption increased fecal acetate concentrations. Avocados are rich in fiber, including soluble hemicelluloses and pectins that can be metabolized by intestinal microorganisms to produce SCFAs (13). *Faecalibacterium* and *Lachnospira* have the enzymatic capability to utilize fibers, such as pectin found in avocados, to form acetate and lactate (57–60). Elevated fiber consumption from pectin-rich apples (61), isolated pectin (60), oat bran (62), and resistant starch (63) have all been shown to increase fecal acetate concentrations. Higher acetate concentrations have also been observed with greater adherence to a Mediterranean diet rich in fiber and MUFAs, nutrients found in high quantities within avocados (64, 65). Acetate is the most prevalent microbial fermentation byproduct, and an energy source for both the microbiota and intestinal epithelial cells through butyrate production. Preclinical studies have demonstrated improved glucose tolerance and reductions in energy intake and body weight with oral SCFA provision (66–69). Increased self-report satiety metrics have also been reported following oral SCFA supplementation among healthy, normal-weight adults (70). Conversely, elevated SCFA concentrations have been observed among adults with overweight and obesity, suggesting aberrations in SCFA production or absorption (7, 8, 71–75). Additional research is required to understand the metabolic role of SCFAs within the context of overweight and obesity.

Avocado intake has been connected to a variety of beneficial health outcomes, including improved lipid profiles and reduced adiposity (15–21); however, to our knowledge, only 1 preclinical trial and 1 human trial to date have reported fecal microbiota findings with avocado consumption. Similar to the present study, a 6-wk rodent trial reported that diets containing 5% and 15% avocado increased fecal acetate concentrations compared with control; however, no differences in bacteria abundances were observed between groups (22). Following a 12-wk hypocaloric dietary intervention involving 51 adults with overweight and obesity, there were some changes in the fecal microbiota (23). Body weight, BMI, and visceral adiposity were reduced in both the control and avocado groups who undertook hypocaloric meal plans with or without 1 Hass avocado/d. Fecal microbial changes included a reduction in Bacteroidetes and an increase in Firmicutes within the avocado group (23), which is similar to the present findings. However, this study did not provide dietary data at post-test and oleic acid concentrations, a nutrient within avocados, did not increase in the avocado group. Additionally, hypocaloric diets have been shown to alter the gut microbiota (76) and thus it is difficult to separate the differences that may be due to caloric restriction from the addition of avocado in that study. The present study is the first, to our knowledge, to evaluate the effects of avocado consumption on the human fecal microbiota in the absence of caloric restriction.

While this randomized controlled trial has many strengths, including having an investigator-blinded study design and provision of study meals that were matched for total energy but varied in nutrient content to match that of the avocado, it is not without limitations. First, while the results demonstrate putative differences in the fecal microbiota between the avocado and control group, there are statistical limitations of these findings, including the exploratory nature of the assessment of the microbiota, which were secondary outcomes of this trial, which do not hold after FDR correction. Participants completed daily meal consumption records and interacted with investigators multiple times per week during the intervention. However, it is possible that the accuracy of self-reported meal consumption and dietary intake records were lacking. Further, participants were adults with overweight and obesity but without physician-diagnosed chronic conditions and, as such, we are unable to generalize our findings to adults within a healthy weight range or individuals with chronic disease.

In conclusion, fresh Hass avocado intake over a 12-wk period resulted in changes to the fecal microbiota and increased concentrations of microbially derived metabolites among adults with overweight and obesity. The fecal bile acid pool was diminished, and relations were observed between fecal bacteria and metabolic biomarkers. These findings provide valuable insight regarding the impact of avocado intake on the intestinal microbiota and have important implications for dietary interventions conducted among the growing at-risk population of adults with overweight or obesity.

## Supplementary Material

nxaa219_Supplemental_File

## Notes

Support for this research was provided by the Hass Avocado Board and the USDA National Institute of Food and Agriculture, Hatch project 1009249. SVT was supported by the USDA National Institute of Food and Agriculture AFRI Predoctoral Fellowship, project 2018–07785, and the Illinois College of ACES Jonathan Baldwin Turner Fellowship. JLK was supported by a Division of Nutrition Sciences Excellence Fellowship. AMT was supported by a Department of Food Science and Human Nutrition Fellowship.

Author disclosures: NAB, NAK, and HDH received grant funding from the Hass Avocado Board.

Supplemental Methods, Supplemental Tables 1–5, and Supplemental Figures 1–4. are available from the “Online Supporting Material” link in the online posting of the article and from the same link in the online table of contents at https://academic.oup.com/jn/.

Abbreviations used: BCFAs, branched-chain fatty acids; BMI, body mass index; CRP, c-reactive protein; FDR, false discovery rate; HOMA-IR, homeostatic model of insulin resistance; IL-6, interleukin 6; MUFAs, monounsaturated fatty acids; PATH, *Persea americana* for Total Health; PERMANOVA, permutational multivariate analysis of variance; QIIME2, Quantitative Insights Into Microbial Ecology version 2; SCFAs, short-chain fatty acids; TNF-α, tumor necrosis factor alpha .
