# Supplementary material for: Avocado Consumption Alters Gastrointestinal Bacteria Abundance and Microbial Metabolite Concentrations among Adults with Overweight or Obesity: A Randomized Controlled Trial
Source: J Nutr. 2020 Aug 17;151(4):753–62. doi: 10.1093/jn/nxaa219 (PMC8030699; doi:10.1093/jn/nxaa219)
Supplement: nxaa219_Supplemental_File [file nxaa219_supplemental_file.pdf]

Avocado consumption alters gastrointestinal bacteria abundance and microbial metabolite concentrations among adults with overweight or obesity: a randomized, controlled trial
Thompson SV *et al.*

Online Supplementary Material

Supplemental Methods

Glucose and insulin analysis: An oral glucose tolerance test was conducted following a 12 hour overnight fast with venous blood collection at baseline, 15, 30, 45, 60, 90, and 120 minutes post ingestion of a beverage containing 250 ml water and 75-gram glucose (NOW Foods, Bloomingdale, Illinois). Blood glucose concentrations were quantified in whole blood samples using a YSI 2900 biochemical analyzer (YSI Life Sciences, Yellow Springs, Ohio) prior to centrifugation and storage at −80°C. Plasma insulin concentrations were quantified using enzyme-linked immunosorbent assays (ELISA, ALPCO, Salem, New Hampshire). Glucose and insulin area under the curve were calculated using fasting, 30, 45, 60, and 120-minute concentrations. The homeostatic model assessment for insulin resistance (HOMA-IR) and the Matsuda index were assessed as surrogate measures of insulin resistance and peripheral insulin sensitivity, respectively (1,2).

Resting Energy Expenditure (REE): REE was determined via indirect calorimetry (Parvo Medics True Max 2400, Salt Lake City, UT) following an overnight fast as previously described (3). Briefly, O_2 usage and CO_2 expiration were analyzed using a calibrated metabolic cart for 45 minutes. REE (kcal/d) was calculated using the Weir equation. A constant physical activity level of 1.3 PAL as presumed to calculate the estimated energy requirements.

Cytokine analysis: Plasma interleukin-6 (IL-6), C-reactive protein (CRP), and tumor necrosis factor (TNF)-alpha, were quantified using ELISA (IL-6, Quantikine HS ELISA [R&D Systems, Minneapolis, MN]; CRP, Human C-reactive protein INSTANT ELISA Kit [ThermoFisher, Carlsbad, CA]; TNF-alpha, TNF-alpha Ultrasensitive ELISA [Alpco, Salem, NH]. Samples were analyzed in duplicate and those with a coefficient of variation in excess of kit-specific cutoffs were repeated or excluded.

Supplementary data

Supplemental Table 1. Per protocol analysis comparisons at 12-week follow-up of fecal bile acid concentrations between groups of adults that consumed a daily isocaloric meal with or without avocado.[1]

	Control Group (n = 44)	Avocado Group (n = 46)	P value
Bile acid, (µg/g dry feces)			
Cholic acid	6.4 ± 2.4	2.4 ± 1.4	0.01
Chenodeoxycholic acid	29.5 ± 9.4	16.4 ± 8.0	0.07
Primary bile acid sum	31.1 ±10.2	16.1 ± 8.0	0.10
Deoxycholic acid	46.4 ± 5.7	36.8 ± 5.9	0.14
Lithocholic acid	43.1 ± 5.2	37.6 ± 3.3	0.73
Secondary bile acid sum	87.3 ± 10.3	72.9 ± 7.9	0.56
Total bile acid sum	115 ± 12.9	88.2 ± 12.9	0.13

[1]Data were analyzed by univariate ANOVA and are presented as means ± standard errors.

Supplementary data

Supplemental Table 2. Per protocol analysis comparisons at 12-week follow-up of fecal fatty acid concentrations between groups of adults that consumed a daily isocaloric meal with or without avocado.[1]

	Control Group (n = 19)	Avocado Group (n = 27)	*P* value[2]	*P* value[3]
Fecal metabolites, (mg/100mg dry feces)				
Palmitic acid (16:0)	$2.14 \times 10^4 \pm 4.83 \times 10^3$	$6.25 \times 10^4 \pm 2.44 \times 10^4$	0.03	0.046
Palmitoleic acid (16:1n-9)	148 ± 36	1510 ± 959	0.01	0.04
Stearic acid (18:0)	$2.35 \times 10^4 \pm 6.61 \times 10^3$	$4.90 \times 10^4 \pm 1.57 \times 10^4$	0.03	0.03
Vaccenic acid (18:1n-11)	974 ± 365	$3.80 \times 10^3 \pm 1.82 \times 10^3$	0.01	0.03
Arachidic acid (20:0)	555 ± 114	$1.22 \times 10^3 \pm 291$	0.04	0.052
Behenic acid (22:0)	450 ± 106	$1.12 \times 10^3 \pm 236$	0.01	0.01
Lignoceric acid (24:0)	275 ± 62	534 ± 88	0.01	0.01

[1]Data were analyzed by univariate ANOVA and are presented as means ± standard errors. [2]P values were not adjusted for dietary intake. [3]P values were adjusted for dietary fatty acid intake obtained from 7-day diet records, excluding lignoceric acid for which total saturated fatty acids was included as a covariate.

Supplementary data

Supplemental Table 3. Intent-to-treat analysis comparisons at 12-week follow-up of dietary intake between group of adults that consumed a daily isocaloric meal with or without avocado.[1]

	Control Group (n = 78)	Avocado Group (n = 78)	*P* value
Total energy (kcal/d)	$2.13 \times 10^3 \pm 69$	$2.31 \times 10^3 \pm 128$	0.33
Total fat (g/d)	88.4 ± 3.3	102.6 ± 6.2	0.053
Saturated fat (g)	31.8 ± 1.1	30.4 ± 1.6	0.11
Monounsaturated fat (g/d)	29.2 ± 1.2	42.8 ± 3.2	<0.001
Polyunsaturated fat (g/d)	20.3 ± 1.0	20.0 ± 1.1	0.62
Total carbohydrate (g/d)	242 ± 8.3	255 ± 14.2	0.73
Total protein (g/d)	81.9 ± 2.8	90.5 ± 5.6	0.23
Total dietary fiber (g/d)	17.2 ± 0.8	27.9 ± 2.5	<0.001
Insoluble dietary fiber (g/d)	11.4 ± 0.5	18.0 ± 1.6	<0.001
Soluble dietary fiber (g/d)	5.6 ± 0.2	9.4 ± 0.9	<0.001
Pectins (g/d)	2.0 ± 0.1	5.1 ± 0.6	<0.001

[1]Data were included for participants with ≥ 5 days of diet records. Diet records were analyzed by univariate ANOVA and are presented as means ± standard errors.

Supplementary data

Supplemental Table 4. Intent-to-treat analysis comparisons at 12-week follow-up of the relative abundance of fecal bacteria between groups of adults that consumed a daily isocaloric meal or and without avocado.[1]

	Control Group (n = 78)	Avocado Group (n = 79)	*P* value	q value
Firmicutes[2]	58.9 ± 1.66	57.5 ± 1.63	0.54	1.20
Blautia[2,4]	4.15 ± 0.49	3.98 ± 0.38	0.85	0.91
Coprococcus[2]	3.42 ± 0.27	3.46 ± 0.25	0.91	0.94
Ruminococcus[2,4]	0.07 ± 0.03	0.10 ± 0.02	0.04	0.39
Dorea[2,4]	1.02 ± 0.11	1.04 ± 0.09	0.82	0.99
Streptococcus[3]	0.58 ± 0.13	0.63 ± 0.15	0.26	0.94
Faecalibacterium[2]	12.8 ± 0.92	15.3 ± 1.03	0.07	0.41
Roseburia[2]	8.13 ± 0.74	6.36 ± 0.48	0.25	1.04
Clostridium[2,4]	0.44 ± 0.06	0.37 ± 0.04	0.44	1.16
Oscillospira[2,4]	1.25 ± 0.13	1.19 ± 0.14	0.74	1.02
Dialister[2,4]	1.69 ± 0.29	2.05 ± 0.33	0.59	1.07
Lactobacillus[3]	0.16 ± 0.07	0.09 ± 0.05	0.56	1.16
Lachnospira[2]	1.11 ± 0.10	1.54 ± 0.15	0.06	0.44
Lachnobacterium[3]	0.26 ± 0.08	0.10 ± 0.05	0.08	0.39
Phascolarctobacterium[2,4]	0.73 ± 0.13	0.69 ± 0.11	0.73	1.06
Alistipes[2,4]	1.50 ± 0.19	2.68 ± 0.37	0.003	0.09
Bacteroidetes[2]	34.7 ± 1.69	35.3 ± 1.59	0.80	1.01
Bacteroides[2]	24.5 ± 1.72	24.0 ± 1.54	0.83	0.93
Parabacteroides[2]	2.44 ± 0.26	2.22 ± 0.23	0.52	1.26
Actinobacteria[2,4]	2.19 ± 0.34	2.01 ± 0.31	0.82	0.95
Bifidobacterium[2,4]	1.51 ± 0.27	1.27 ± 0.23	0.64	1.09
Collinsella[3]	0.37 ± 0.14	0.38 ± 0.11	0.56	1.08
Verrucomicrobia[3]	1.05 ± 0.24	1.71 ± 0.55	0.30	0.87
Akkermansia[3]	1.56 ± 0.52	1.19 ± 0.32	0.66	1.01
Proteobacteria[2]	2.74 ± 0.28	2.78 ± 0.27	0.91	0.91
Sutterella[2,4]	1.61 ± 0.22	1.98 ± 0.23	0.26	0.84
Bilophila[2,4]	0.27 ± 0.04	0.32 ± 0.05	0.65	1.05
Desulfovibrio[3]	0.16 ± 0.05	0.12 ± 0.04	0.78	1.03

[1]Data were analyzed by univariate ANOVA[2] or Mann-Whitney U test[3] with False Discovery Rate correction and are presented as means ± standard errors. [4]Indicates bacteria that were transformed prior to analysis.

Supplementary data

Supplemental Table 5. Intent-to-treat analysis comparisons at 12-week follow-up of fecal bile acid concentrations between groups of adults that consumed a daily isocaloric meal with or without avocado.[1]

	Control Group (n = 62)	Avocado Group (n = 63)	*P* value
Bile acid, (µg/g dry feces)			
Cholic acid	6.7 ± 2.1	5.6 ± 2.8	0.01
Chenodeoxycholic acid	29.5 ± 8.4	31.4 ± 14.5	0.19
Primary bile acid sum	32.0 ± 9.3	30.0 ± 14.0	0.07
Deoxycholic acid	48.4 ± 4.8	36.1 ± 5.0	0.08
Lithocholic acid	44.3 ± 4.1	35.9 ± 2.7	0.09
Secondary bile acid sum	91.1 ± 8.2	69.0 ± 6.7	0.10
Total bile acid sum	120 ± 11.7	97.5 ± 14.7	0.07

[1]Data were analyzed by univariate ANOVA and are presented as means ± standard errors.

Supplementary data

Supplemental Figure 1.

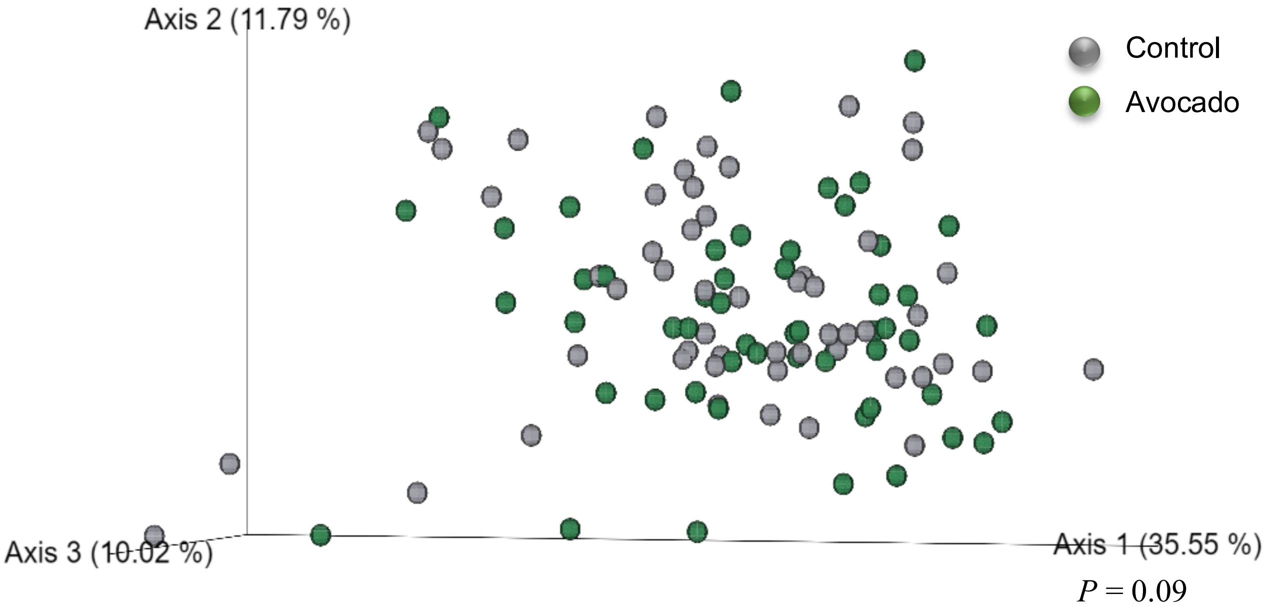

Per protocol analysis comparisons at 12-week follow-up of weighted Unifrac distances between groups of adults that consumed a daily isocaloric meal with or without avocado. Sequence data were rarefied to 6,652 sequences per sample prior to comparisons of the Weighted Unifrac distances between the avocado (n=55) and control (n=54) groups. The results revealed that the groups tended (*P*=0.09) to differ from each other.

Supplementary data

Supplemental Figure 2.

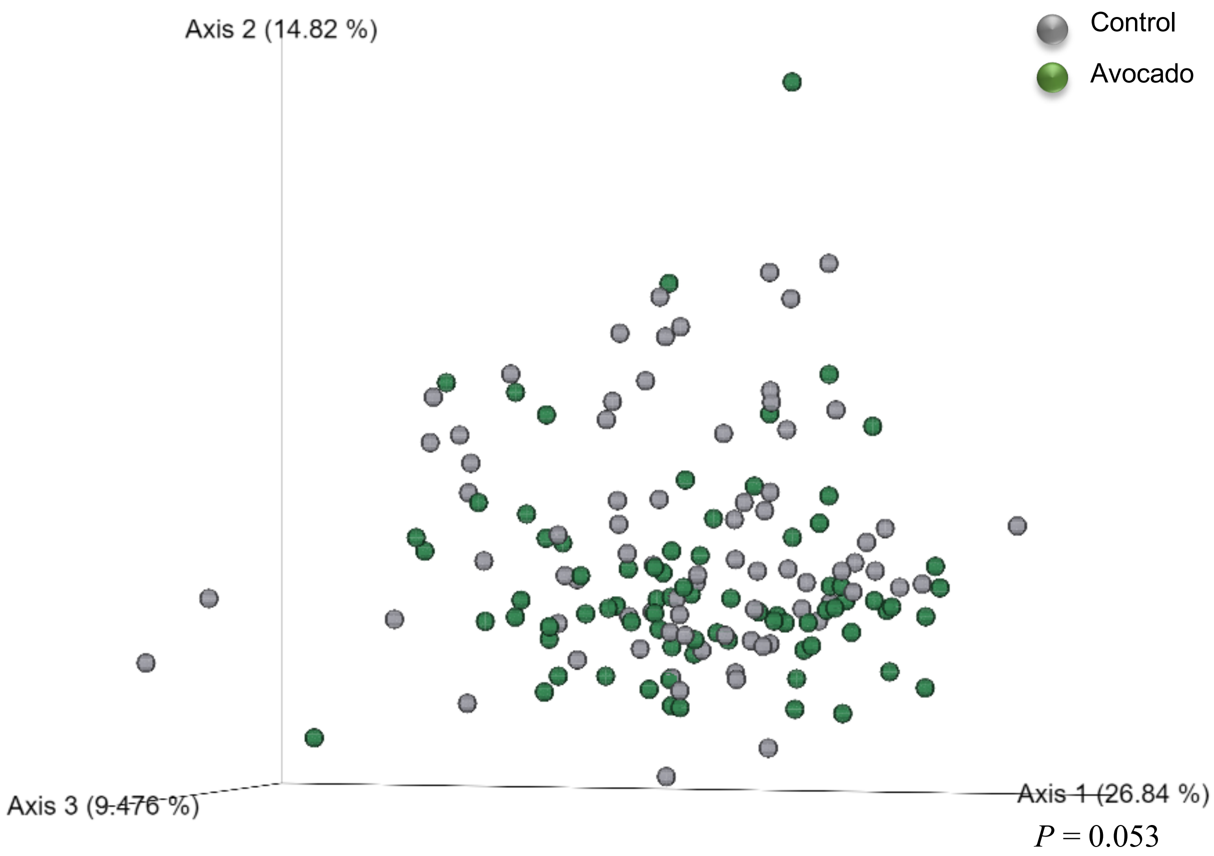

Intent-to-treat analysis comparisons at 12-week follow-up of the weighted Unifrac distances between groups of adults that consumed a daily isocaloric meal with or without avocado. Sequence data were rarefied to 6,450 sequences per sample prior to comparisons of the Weighted Unifrac distances between the avocado and control groups. The results revealed that the groups tended (P=0.053) to differ from each other.

Supplementary data

Supplemental Figure 3.

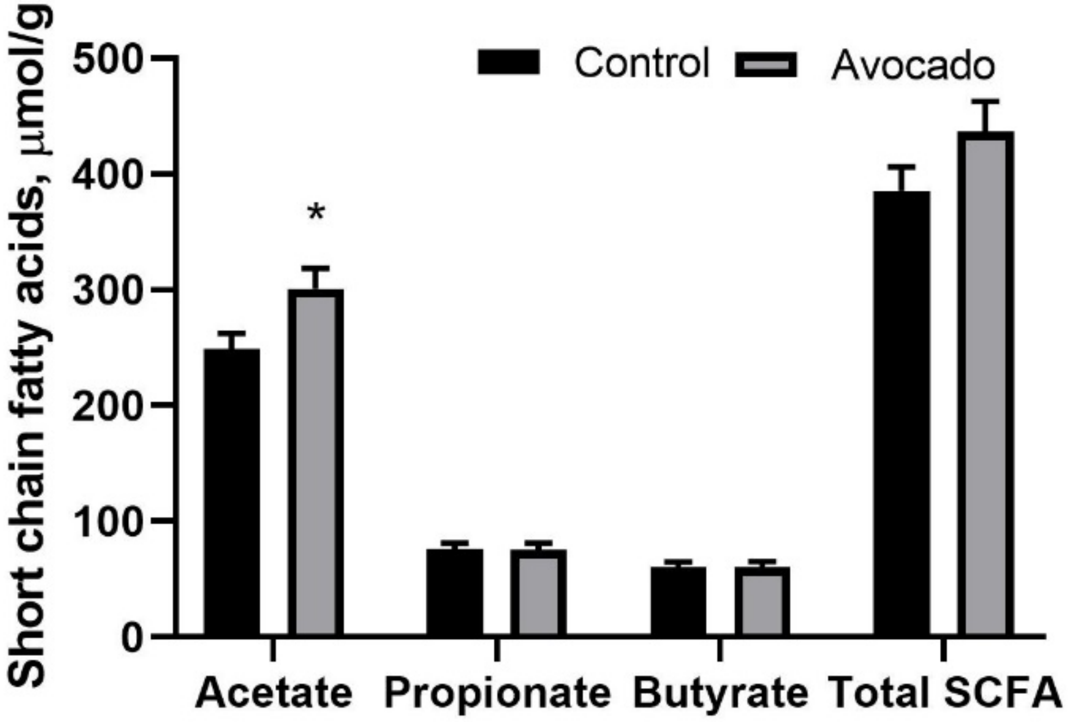

Intent-to-treat analysis comparisons at 12-week follow-up of fecal short chain fatty acid concentrations between groups of adults that consumed a daily isocaloric meal with or without avocado. Data were analyzed by univariate ANOVA and are presented as mean ± standard errors (n = 78 Control, n = 79 Avocado). *Different from Control, $P < 0.05$.

Supplementary data

Supplemental Figure 4.

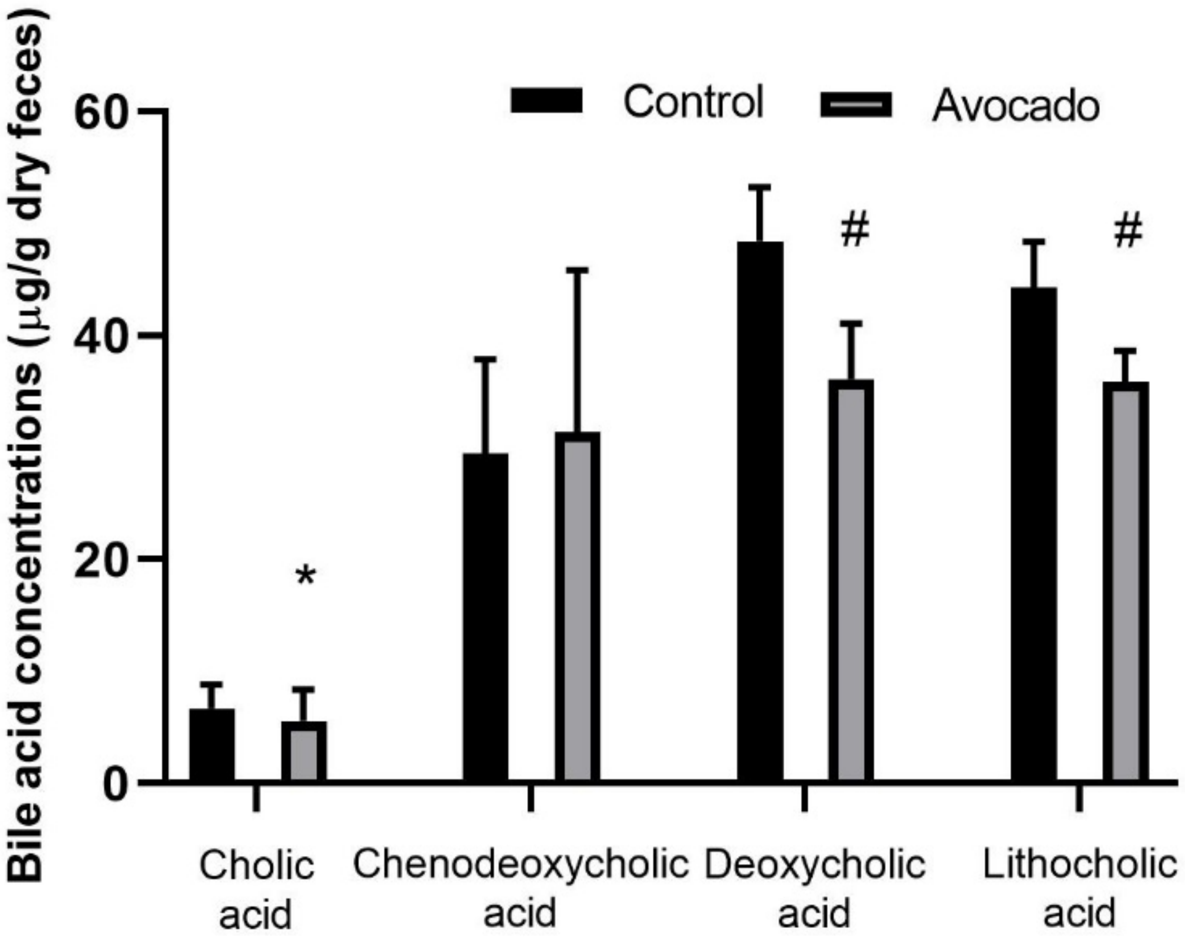

Intent-to-treat analysis comparisons at 12-week follow-up of fecal primary and secondary bile acid concentrations between groups of adults that consumed a daily isocaloric meal with or without avocado. Data were analyzed by univariate ANOVA and are presented as mean ± standard errors (n = 62 Control, n = 63 Avocado). *Different from Control, $P < 0.05$; #tended to differ from Control $P < 0.1$.

Supplementary data

Supplemental References

1. Matsuda M, DeFronzo RA. Insulin sensitivity indices obtained from oral glucose tolerance testing: comparison with the euglycemic insulin clamp. Diabetes Care. 1999;22:1462–70.

2. Matthews DR, Hosker JP, Rudenski AS, Naylor BA, Treacher DF, Turner RC. Homeostasis model assessment: insulin resistance and beta-cell function from fasting plasma glucose and insulin concentrations in man. Diabetologia. 1985;28:412–9.

3. Hannon BA, Edwards CG, Thompson S V, Reeser GE, Burd NA, Holscher HD, Teran-Garcia M, Khan NA. Single nucleotide polymorphisms related to lipoprotein metabolism are associated with blood lipid changes following regular avocado intake in a randomized control trial among adults with overweight and obesity. J Nutr. 2020;6:1379-1387
